# Validation of the Arabic Version of the Early Childhood Oral Health Impact Scale (ECOHIS)

**DOI:** 10.1186/s12903-017-0353-x

**Published:** 2017-02-28

**Authors:** Nada J. Farsi, Azza A. El-Housseiny, Deema J. Farsi, Najat M. Farsi

**Affiliations:** 1 Department of Dental Public Health, Faculty of Dentistry, King Abdulaziz University, P.O. Box 80200, Jeddah, 21589 Saudi Arabia; 2 Department of Pediatric Dentistry, Faculty of Dentistry, King Abdulaziz University, Jeddah, Saudi Arabia; 3 Department of Pediatric Dentistry, Faculty of Dentistry, Alexandria University, Alexandria, Egypt

**Keywords:** Quality of life, Validation, ECOHIS, Preschool children

## Abstract

**Background:**

Assessment of the adverse effects of oral health problems on oral health-related quality of life (OHRQoL) is essential to ensure the well-being of children. The Early Childhood Oral Health Impact Scale (ECOHIS) is an instrument that was designed to assess caregivers’ perceptions of OHRQoL in preschool children. Although it has been translated into many languages, it has yet to be validated in Arabic. Therefore, this study aimed to translate this questionnaire to Arabic (A-ECOHIS) and test its psychometric properties.

**Methods:**

Questionnaire responses from three samples of caregivers of preschool children ≤ 6 years of age were collected: (i) community-based (*n* = 422), from preschools selected as a stratified random sample; (ii) clinic-based, from those seeking pediatric dental care at a university clinic (*n* = 246); and (iii) a test-retest sample (*n* = 68), a clinic-based group of caregivers who completed questionnaires twice about siblings who were not receiving dental care. Children received a dental examination to assess their decayed, missed, filled teeth (dmft) scores. Convergent validity was evaluated by assessing the A-ECOHIS scores in relation to the response to a global question. Discriminant validity was evaluated by comparing the scores of children with varying levels of oral disease. Internal consistency was assessed by calculating Cronbach’s alpha, and the test-retest reliability was assessed using intra-class correlation coefficients (ICCs).

**Results:**

The A-ECOHIS scores of the questionnaire sections and the global oral health rating were significantly correlated; Spearman correlation coefficients were, r = 0.55, P ≤ 0.01 (overall score), r = 0.54, P ≤ 0.01 (child section), and r = 0.51, P ≤ 0.01 (family section). The mean A-ECOHIS scores were also statistically significantly higher in children with higher dmft scores compared with lower dmft, and in the clinic-based sample compared with the community sample. The Cronbach’s alpha value of the the child, family sections and overall questionnaire were, 0.80, 0.78, and 0.85, respectively. The intra-class correlation coefficient (ICC) of A-ECOHIS was 0.86.

**Conclusion:**

The A-ECOHIS performed well on all psychometric tests to which it was applied. Thus, it is a valid and reliable instrument that can be used in Arabic-speaking caregivers of preschoolers aged 2 to 6 years.

## Background

Children younger than six years of age are especially vulnerable to oral health problems [1]. These problems include temporary teething discomfort, trauma to the teeth and supporting structures, and early childhood caries [2]. Caries, despite recent preventive regimes and advanced early diagnosis methods, remains a prevalent childhood disease around the world. In fact, dental caries is the most common chronic disease in children [3]. In the US, 1 in every 4 children between 2 and 5 years of age has had caries in the primary teeth [4]. The prevalence of caries is also high in most Arabi countries including Saudi Arabia [5, 6]. In Saudi Arabia, the prevalence of caries was estimated to be 73% [7], and a meta-analysis by Khan et al. found a mean decayed, missing, filled teeth (dmft) score due to caries of 5.38 in primary dentition [8].

Oral health problems can have a negative effect on a young child’s functional, psychological, and social well-being and, in turn, on the family as a whole. The impact of such problems can be manifested as pain, loss of function, psychological effects, problems with proper weight gain and growth, restriction of daily activities, and disruption of the normal family functioning [9–12].

Although clinical manifestations are of pivotal importance, the physical and psychosocial impact of oral disease cannot be assessed by these parameters alone [13]. Recently, healthcare professionals have begun to incorporate into the oral health assessment the patients’ perceptions of how their oral health affects their quality of life (QoL) [14].

A number of oral health-related QoL (OHRQoL) instruments have been designed to assess the impact of oral health problems, some of which focus on the pediatric population. These include the Parental-Caregiver Perceptions Questionnaire (P-CPQ) [15], the infantile and toddler QoL (ITQoL), child oral health impact profile (COHIP), child oral impact daily performance (child-OIDP), child perception questionnaire (CPQ) [16–20] and Caries-QC [21]. However, it was not until 2007, when Pahel et al. developed the Early Childhood Oral Health Impact Scale (ECOHIS), that an instrument was designed especially for young children [22].

The ECOHIS tests the impact of oral health problems on both young children and their families. Because preschoolers have not reached a developmental and psychological level that allows them to accurately recall past events and give accurate accounts of personal experiences, the questionnaire is designed for adult caregivers, who can better relate the impact of oral health on the child’s life [22].

The ECOHIS has performed well and has shown good reliability and validity. The scale has been translated into several languages and has been tested and validated on diverse populations with promising results [23–28]. The first translation was into French [23], followed by Chinese [25], Brazilian Portuguese [24, 27], Spanish [26], Lithuanian [29], and Malay [28]. In the Middle East, it has been translated into Farsi and Turkish [30, 31]. In this study, we aimed to translate ECOHIS into the Arabic language (A-ECOHIS) and test its psychometric properties on an Arabic-speaking population.

## Methods

### The questionnaire

The original ECOHIS questionnaire was developed in English by Pahel et al., who demonstrated its validity and reliability [22]. It comprises 13 questions and is divided into child and family impact sections. The child impact section includes nine items and comprises four domains: child symptoms, function, psychology, and self-image and social interaction. The family impact section contains four items and comprises two domains: parental distress and family function.

### Development of A-ECOHIS

**Fig. 1:**
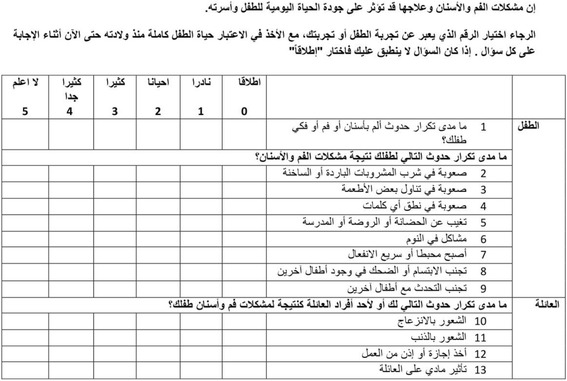
The Arabic version of the Early Childhood Oral Health Impact Scale (A-ECOHIS)

The English version of the ECOHIS was translated into Arabic using the well-recognized forward-backward translation technique [32]. Two native Arabic speakers, who speak English fluently, independently translated the original English version of ECOHIS. The Arabic versions were revised with the aid of one of the authors and the two translators, from which one preliminary Arabic version was produced. This version was translated back to English by two bilingual professionals. Finally, the two back-translated English versions were compared with the original English version, and minor adjustments were made to the final Arabic version by adjusting the translation of the words “trouble” and “upset” in questions 6 and 10, respectively (Fig. 1). As with the English version [22], response options for A-ECOHIS were coded as follows: 0 = never, 1 = hardly ever, 2 = occasionally, 3 = often, 4 = very often, and 5 = don’t know; and subjects were asked to answer the questions based on the whole life span of the child. To test the questionnaire’s comprehensibility, a pilot study was conducted with 10 caregivers not involved in the main study, and the questionnaire was simplified.

The total score was calculated by summing the scores of all questions, and separate scores were calculated for each of the questionnaire sections. The range of the child section scores was 0 to 36, and the range of the family section was 0 to 16. Missing values were handled as described in the original study [22]; “don’t know” responses were treated as missing. For maximum utilization of the data, for subjects with ≤ 2 missing values in the child impact section, or ≤ 1 missing value in the family impact section, the missing values were imputed by using the mean of the rest of the values of each section accordingly. Therefore, subjects could be included in the analysis of one section but not the other, as was done in the original study [22]. Subjects with > 2 missing values in the child impact section and with > 1 missing value in the family impact section were excluded.

### Study subjects

In this study, community-based and clinic-based samples were collected. For participants to be included, Arabic had to be the native language of the caregivers, and they had to be able to fill in the questionnaire independently. They also had to be living with the child for ≥ 50% of the time. Their children had to be healthy, not on long-term medications, and with no physical, learning, or mental disabilities. The participants of the community-based sample (*n* = 425) were caregivers of preschool children ≤ 6 years of age. The targeted population included all Saudi and non-Saudi children who were registered in kindergarten schools in Jeddah according to the Ministry of Education (population, 14,808 children). The sampling method of the study was multistage stratified random sampling from the preschool children in Jeddah. There were 7448 male and 7360 female children distributed among 34 public and 181 private preschools.

It was determined to choose the school as a unit of sample selection using a numbered list that was previously prepared. Preschools were randomly selected using Random Number Generator [33]. Two numbers representing a private and a public school were selected in each of Jeddah’s main four districts (North, East, South, and West). Approval to visit the selected preschools and collect data from the children was obtained from the Ministry of Education in Jeddah, and approval of the school principal was obtained prior to the school visit.

During the first preschool visit, consent forms with information about the study were distributed to the children. Children were encouraged to bring back the signed consent forms the next morning. At the next school visit, examinations were performed on the children who brought back a signed consent.

The clinic-based group (*n* = 246) comprised caregivers of a convenience sample of children 6 years old or younger seeking dental care in the pediatric dental clinics of King Abdulaziz University. The A-ECOHIS was given to the participating caregivers for completion while they were at the clinic. Socio-demographic information on the caregivers and children was also collected. In total, 750 questionnaires were distributed, and the response rate was 89.5%. The recruitment period was from September 2013 to April 2014.

Children in the community and clinic-based samples received a dental examination by one examiner who used an agreed-upon rubric and was trained and calibrated in the Faculty of Dentistry of King Abdulaziz University for the detection of caries in 2 to 6 year-old children using the World Health Organization 1997 criteria [34].

Children were seated on a chair with a back rest; a knee to knee position was used with very young children. Caries experience as decayed, missed and filled teeth (dmft) were diagnosed using a blunt Community Periodontal Index (CPI) probe (Nordent, Elk Grove Village, IL, USA), disposable plane mirror and adequate light using standard infection control measures. Teeth were examined visually and the CPI probe was used to remove debris and confirm visual evidence of caries. Teeth were recorded as sound, if they showed no evidence of treatment or caries into dentin. Teeth with white, discolored or rough spots, stained pits or fissures without cavitations or softening were also recorded as sound. Teeth were recorded carious, if there was a visible cavity, undermined enamel or detectably softened floor or wall. Teeth only extracted due to caries were recorded as missing [34]. A confidential report was given to the caregivers of the community-based sample with advice to seek dental care in any dental facility if needed.

Ethical approval for the study was obtained from the Research Ethics Committee, Faculty of Dentistry King Abdulaziz University (#036-13). Informed consent was obtained from all participating caregivers.

### Validation of A-ECOHIS

#### Convergent validity

To test the convergent validity for the community-based sample, the following global oral health question was added: “In general, how would you rate the overall oral health of your child?” This question had five response options: 0 = excellent, 1 = very good, 2 = good, 3 = fair, and 4 = poor. The mean A-ECOHIS scores of each group of respondents, according to their global question response were compared. We hypothesized that caregivers with a response of “excellent” on the global question would have a low A-ECOHIS score, and that the score would increase as the global question responses became lower. Furthermore, ratings of “excellent”, “very good” and “good” in the global question were combined into “good health”, while “fair” and “poor” ratings were combined into “poor health”, and the mean A-ECOHIS scores for both levels were compared with a *t*-test, which is robust against non-normality when the sample size is ≥ 40 [35].

Spearman’s rank order correlations were used to assess correlations between the responses to the global question and each of the A-ECOHIS section scores.

#### Discriminant validity

In the community-based sample, the A-ECOHIS scores for each of the sections between children with varying dmft scores (none, 1–5, >5) were compared. We hypothesized that children with higher dmft scores would have higher A-ECOHIS scores. To further assess the discriminant validity of A-ECOHIS, we compared the A-ECOHIS scores of each questionnaire domain between the community-based and clinic-based samples. Because children in the clinic-based sample probably had worse oral health, we hypothesized that they would have lower A-ECOHIS scores.

### Internal consistency

Spearman’s rank order correlations were used to assess the correlations between the child and family impact section scores in the community-based sample, and to estimate the inter-item correlations. Cronbach’s alpha was also produced for each of the scale sections and for the whole scale. We hypothesized that the child and family scores as well as the scale items would be correlated.

### Test-retest reliability

A sample was collected (*n* = 78) to assess the test-retest reliability of the questionnaire. The caregivers of children seeking treatment at the Pediatric Dental Clinics in King Abdulaziz University, who were accompanied by siblings aged 0 to 6 years, were approached, and consenting caregivers were asked to complete the questionnaire about the sibling who was not receiving dental treatment. They were given the questionnaire again after 2 to 3 weeks, when they came for a follow-up appointment. It was verified that the participating subjects did not report changes in their child’s oral health condition or treatments during this period. The intra-class correlation coefficient was used to assess the test-retest reliability in the sample for each of the questionnaire sections and for the questionnaire as a whole.

### Sample size calculation

Using the tables developed by Saunders and Huynh, a sample size of 245 subjects was determined for reliability testing [36], given the assumption that the ECOHIS is a 13-item test of a moderate degree of difficulty and low variability. The calculation was made considering the degree of precision to be 0.05%.

## Results

Of the 425 community-based participants, three subjects who had missing values for more than two items of the child section and more than one item of the family section were excluded from the analyses. One participant of the clinic sample with more than 2 missing values in the child section and no missing values in the family section was included in the score calculation for the family section, but not the child section. In addition, 20 participants from the community sample with more than 1 missing value in the family section and no missing values in the child section were included in the child section analyses only.

Table 1 presents the demographic characteristics of the participants. The mean age of the community-based sample was 4.5 ± 0.6 years, whereas the mean age of the clinic-based sample was 4.6 ± 1.2 years. Females comprised 57 and 52% of the community and clinic samples, respectively. Of the community sample, 81% attended private schools, while 19% attended public schools. The clinic sample had statistically significantly higher dmft scores compared with the community sample (9.9 ± 5.0 and 4.0 ± 4.4, respectively).

**Table 1:** Demographic characteristics of the study participants

Variable	Community-based sample	Clinic-based sample	*P*-Value
	*n* = 422 (%)	*n* = 246 (%)	
Age, mean (SD)	4.5 (0.6)	4.6 (1.2)	0.246
Gender			
Male	181 (42.9)	117 (47.6)	0.242
Female	241 (57.1)	129 (52.4)	
Nationality			
Saudi	263 (63.2)	123 (50.0)	<0.01
Non-Saudi	153 (36.8)	123 (50.0)	
School type			
Private	343 (81.3)	--	--
Public	79 (18.7)	--	
dmft score			
0	136 (32.2)	11 (4.5)	<0.01
1-5	161 (38.2)	40 (16.3)	
> 5	125 (29.6)	195 (79.3)	
dmft score, mean (SD)	4.0 (4.4)	9.9 (5.0)	<0.01

*SD* standard deviation, *dmft* decayed, missing, filled teeth

The distribution of the A-ECOHIS responses is presented in Table 2. Among the community-based subjects, pain was the most reported item (35%), followed by irritability or frustration (24%), and difficulty eating (24%), in the child section. Caregivers being upset was the most reported item in the family section (31%). There was one response of “don’t know” to three items (pain, irritability or frustration, and avoid talking) and missing values, which ranged from 0.2% for the pain item to 5.5% for the financial impact item. Among the clinic-based subjects, in the child section, the most commonly reported item was pain (65%), followed by eating difficulty (57%), and difficulty in drinking hot or cold drinks (46%). Most commonly reported in the family section was a caregiver being upset (65%), followed by feeling guilty (53%) about the child’s perceived oral health condition. It was evident that subjects in the clinic sample were experiencing a greater impact on QoL compared to those in the community sample. There were 2% of subjects who answered “don’t know” to the school absence item, whereas 0.8% reported this for the pronunciation difficulty, avoid smiling or laughing, and avoid talking items. Missing values ranged from 0.4% for feeling guilty, and for irritability and frustration, to 2.8% for the school absence item.

**Table 2:** Distribution of A-ECOHIS responses in the two study samples

	Community-based sample (*n* = 422)			Clinic-based sample (*n* = 246)		
	Never/hardly ever n (%)	Occasionally, Often, Very often n (%)	Don’t know n (%)	Never/hardly ever n (%)	Occasionally, Often, Very often n (%)	Don’t know n (%)
I] Child impact section						
*i) Symptoms*						
Q1 Pain	274 (64.9)	147 (34.8)	1 (0.2)	86 (35.0)	160 (65.0)	0
*ii) Function*						0
Q2 Difficulty drinking hot or cold beverages	337 (80.2)	83 (19.8)	0	132 (53.7)	114 (46.3)	0
Q3 Difficulty eating	319 (76.1)	100 (23.9)	0	105 (43.2)	138 (56.8)	
Q4 Pronunciation difficulty	351 (84.2)	66 (15.8)	0	193 (78.8)	50 (20.4)	2 (0.8)
Q5 Missed school or daycare	346 (82.4)	74 (17.6)	0	195 (79.9)	44 (18.0)	5 (2.1)
*iii) Psychology*						
Q6 Trouble sleeping	368 (88.5)	48 (11.5)	0	178 (73.3)	65 (26.8)	0
Q7 Irritability or frustration	317 (75.3)	103 (24.5)	1 (0.2)	172 (70.2)	73 (29.8)	0
*iv) Self-image and social interaction*						
Q8 Avoid smiling or laughing	378 (90.2)	41 (9.8)	0	199 (81.6)	43 (17.6)	2 (0.8)
Q9 Avoid talking	367 (89.5)	42 (10.2)	1 (0.2)	205 (84.0)	37 (15.2)	2 (0.8)
II] Family impact section						
*i) Parental distress*						
Q10 Been upset	286 (69.4)	126 (30.6)	0	85 (34.8)	159 (65.2)	0
Q11 Felt guilty about child’s oral health	329 (79.9)	83 (20.2)	0	116 (47.4)	129 (52.7)	0
*ii) Family function*						
Q12 Taken time off work	351 (87.3)	51 (12.7)	0	154 (63.4)	89 (36.6)	0
Q13 Financial impact	345 (86.5)	54 (13.5)	0	165 (67.1)	81 (32.9)	0

*A-ECOHIS* Arabic version of the Early Childhood Oral Health Impact Scale

Convergent validity was assessed using the community-based data. The mean A-ECOHIS overall score was much lower for subjects who responded “excellent” (3.9 ± 4) compared with those who responded “poor” (19.1 ± 6.8) to the global health rating question. Similar trends were observed in the child and family sections. When responses to the global question were dichotomized into good and poor health, A-ECOHIS scores in the scale overall and in both of its sections were statistically significantly higher in the latter. The Spearman correlation coefficients between the global oral health rating and the total A-ECOHIS score (r = 0.55, P ≤ 0.01), as well as those for the child (r = 0.54, P ≤ 0.01) and family section (r = 0.50, P ≤ 0.01) scores, were moderate (Table 3).

**Table 3:** Evaluating the difference in mean A-ECOHIS scores by oral health status rating category

Global health rating question response	Child impact section				Family impact section				Overall scale			
	n	Mean (SD)	r^a^	*P*-value	n	Mean (SD)	r^b^	*P*-value	n	mean (SD)	r^c^	*P*-value
Correlations												
Excellent	147	2.85 (3.3)	0.54	--	146	1.05 (1.8)	0.50	<0.01	146	3.87 (4.1)	0.55	--
Very good	144	4.96 (3.7)			140	2.63 (2.6)			140	7.56 (5.2)		
Good	57	8.04 (4.9)			57	4.56 (3.6)			57	12.60 (7.6)		
Fair	43	8.92 (4.7)			31	3.39 (2.5)			31	10.85 (5.5)		
Poor	22	12.62 (5.5)			21	6.21 (2.6)			21	19.14 (6.8)		
Differences in mean scores												
Good oral health	348	4.57 (4.1)	--	<0.01	343	2.28 (2.8)	--	<0.01	343	6.83 (6.1)	--	<0.01
Poor oral health	65	10.17 (5.2)			52	4.53 (2.9)			52	14.20 (7.3)		

*A-ECOHIS* Arabic version of the Early Childhood Oral Health Impact Scale

Excluded from this analysis were 27 subjects who had > 1 missing item on the family section (*n* = 18), missing information on the global question (*n* = 7), and missing both > 1 item on family section and information on the global question (*n* = 2). Spearman correlation coefficients between ^a^child impact section score and the global question, ^b^family impact section score and the global question, and ^c^total A-ECOHIS score and the global question

To test discriminant validity, the A-ECOHIS scores among the community-based data for each of the questionnaire sections were stratified by dmft score (Table 4). The mean A-ECOHIS scores of each section were higher in the higher dmft groups. However, the differences between dmft scores of 0 and 1–5 were not statistically different in some of the domains. The mean A-ECOHIS scores for subjects with a dmft score of zero in the child section, family section and total score were 4.2 ± 4.2, 2.1 ± 2.7, and 6.3 ± 6.2, respectively, whereas they were 4.7 ± 4.3, 2.2 ± 2.9, and 6.6 ± 5.9, respectively, among participants with a dmft score of 1–5. Among subjects with a dmft above 5, the A-ECOHIS scores were 7.8 ± 5.0, 3.6 ± 3.0 and 11.2 ± 7.1, respectively.

**Table 4:** Discriminate validity of the A-ECOHIS among the community-based sample

	Number of items	Range	Number of decayed, missed and filled teeth			Multiple ANOVA comparisons
			None	1–5	>5	
Child symptoms	1	0–4				0 vs. 1–5*
Sample size			136	161	125	1–5 vs. ≥5*
Mean score (SD)			0.5 (0.8)	0.9 (1.0)	1.6 (0.9)	0 vs. ≥5*
Child function	4	0–16				0 vs. 1–5
Sample size			136	161	125	1–5 vs. ≥5*
Mean score (SD)			2.1 (2.1)	2.1 (2.2)	3.7 (2.7)	0 vs. ≥5*
Child Psychology	2	0–8				0 vs. 1–5
Sample size			136	161	125	1–5 vs. ≥5*
Mean score (SD)			1.0 (1.4)	1.0 (1.3)	1.5 (1.5)	0 vs. ≥5*
Self-image and social interaction	2	0–8				0 vs. 1–5
Sample size			136	161	125	1–5 vs. ≥5*
Mean score (SD)			0.6 (1.2)	0.6 (1.1)	1.1 (1.5)	0 vs. ≥5*
*Child impact section*	9	0–36	4.2 (4.2)	4.7 (4.3)	7.8 (5.0)	0 vs. 1–5
						1–5 vs. ≥5*
						0 vs. ≥5*
Parental Distress	2	0–8				0 vs. 1–5
Sample size			134	153	115	1–5 vs. ≥5*
Mean score (SD)			1.2 (1.7)	1.4 (1.8)	2.3 (2.0)	0 vs. ≥5*
Family function	2	0–8				0 vs. 1–5
Sample size						1–5 vs. ≥5*
Mean score (SD)			134	153	115	0 vs. ≥5*
			0.9 (1.4)	0.8 (1.5)	1.4 (1.6)	
*Family impact section*	4	0–16	134	153	115	0 vs. 1–5
			2.1 (2.7)	2.2 (2.9)	3.6 (3.0)	1–5 vs. ≥5*
						0 vs. ≥5*
*Overall scale*	13	0–52	6.3 (6.2)	6.6 (5.9)	11.2 (7.1)	0 vs. 1–5
						1–5 vs. ≥5*
						0 vs. ≥5*

*A-ECOHIS* Arabic version of the Early Childhood Oral Health Impact Scale, *SD* standard deviation

Excluded from the family section and total ECOHIS analyses were 20 subjects with > 1 missing item in the family section. *P-value ≤ 0.05, Tukey multiple comparisons test

When the A-ECOHIS scores were compared between the two samples, the A-ECOHIS mean scores were significantly higher among the clinic-based subjects for each of the questionnaire domains (Table 5). In the community sample, floor effects were observed in 19 and 40% of the participants in the child and family sections, respectively. However, they were observed only in 13% each of the child and family sections in the clinic sample. No ceiling effects were observed in either of the questionnaire sections in the community sample, and they were observed only in the family section in 2.9% of the clinic-based subjects (data not shown).

**Table 5:** Comparison of A-ECOHIS scores of the different domains in the two study samples

Impacts	Number of items	Range	Community-based sample			Clinic-based sample			*P*-value*
			Mean ± SD	Median	Floor effects	Mean ± SD	Median	Floor effects	
Child symptoms	1	0–4	1.0 (1.0)	1	42%	2.0 (1.3)	2	17%	<0.01
Child function	4	0–16	2.6 (2.4)	2	28%	4.3 (3.4)	4	20%	<0.01
Child Psychology	2	0–8	1.1 (1.4)	0	51%	1.9 (2.0)	1	38%	<0.01
Self-image and social interaction	2	0–8	0.8 (1.3)	0	66%	1.0 (1.7)	0	63%	0.018
Child impact section	9	0–36	5.5 (4.7)	5	19%	9.2 (7.1)	8	13%	<0.01
Parental Distress	2	0–8	1.6 (1.9)	1	44%	3.5 (2.3)	4	17%	<0.01
Family function	2	0–8	1.0 (1.5)	0	58%	2.1 (2.1)	2	34%	<0.01
Family impact section	4	0–16	2.6 (2.9)	2	40%	5.5 (3.9)	5	13%	<0.01
Overall scale	13	0–52	7.8 (6.7)	7	16%	14.6 (9.2)	14	3%	<0.01

*A-ECOHIS* Arabic version of the Early Childhood Oral Health Impact Scale, *SD* standard deviation

*t-test was used

In the community-based sample, the Spearman correlation coefficient for the relationship between the child and family section scores was of a moderate magnitude but was statistically significant (r = 0.56, P ≤ 0.01). As presented in Table 6, Cronbach’s alpha values for the child section, family section, and overall questionnaire were 0.80, 0.78, and 0.85, respectively. The inter-item correlations between the 13 items of the A-ECOHIS ranged from 0.1 to 0.7; all were positive and statistically significant (P ≤ 0.01–0.03).

**Table 6:** Reliability analyses of the A-ECOHIS: internal consistency and test-retest reliability (*n* = 68)

Impacts	Internal consistency reliability (Cronbach’s alpha)	Test-retest reliability ICC (95% CI)
Child impact section	0.80	0.89 (0.82–0.93)
Family impact section	0.78	0.67 (0.52–0.79)
Overall scale	0.85	0.86 (0.78–0.91)

*A-ECOHIS* Arabic version of the Early Childhood Oral Health Impact Scale, *ICC* intraclass correlation coefficient, *95% CI* 95% confidence interval

Test-retest reliability was assessed on the sample of subjects who were administered the questionnaire twice (*n* = 78); however, 10 of these subjects were excluded because of missing data. The pre-test A-ECOHIS scores of the child and family sections were 6.8 ± 6.5, and 5.1 ± 4.2, respectively, and were 7.4 ± 7.1 and 6.3 ± 4.2, respectively, at the post-test. The mean of the total A-ECOHIS score was 11.9 ± 9.3 at the pre-test and 13.7 ± 10.3 at the post-test. The estimated intra-class correlation coefficients are presented in Table 6.

## Discussion

Oral health problems can have a negative impact on the OHRQoL of children and their families [22]. Evaluating and describing these influences can aid dentists in assessing children’s oral health needs [11, 24, 25, 30, 37], providing better oral health care services [11, 24, 26, 31], and improving the oral health outcomes of children [11, 22]. Although assessment of OHRQoL is well established in adults following several decades of use, its application in children is relatively new and less understood. Prior to Pahel et al’s development of ECOHIS [22], the first documented child-specific OHRQoL scale was presented by Jokovic et al. in 2002 to assess the impact of oral conditions on QoL in children 11 to 14 years of age [38]. Proper translation and validation are important for cross-cultural adaptation of QoL questionnaires [24–26, 39] and also enable comparisons between different countries using the same measurement instrument [24–26, 31, 40]. The ECOHIS questionnaire has so far been translated into French [23], Chinese [25], Farsi [30], Turkish [31], Brazilian Portuguese [24, 27], Spanish [26], Lithuanian [29], and Malay [28]. In this study, the ECOHIS was translated into the Arabic language, and its psychometric properties were tested.

Pain was the most frequently reported negative impact measure in the child section in both of our samples, and was common in other studies [22–25, 27, 28]. However, in the Turkish [31] and Lithuanian [29] studies, difficulty in eating and irritability, respectively, were most commonly reported. Similar to some studies [23–25, 29, 31], caregivers feeling upset was the most frequently reported item in the family section in this study. Taking time off work [22] and feeling guilty [27, 28] were the most commonly reported family impacts in three other studies. Although treatment at the clinic from which our sample was recruited is provided free of charge, about a third of our clinic-based caregivers reported a financial impact, which could have been attributed to other expenses incurred, such as transportation costs or missing work.

Floor effects are expected in community-based samples because only a small percentage of subjects have oral diseases [41], which explains the higher rate of floor effects among the community sample in this study. Accordingly, in the original scale study [22] and in its French [23] and Spanish translations [26], heavy floor effects were observed. The Turkish [31], Chinese ECOHIS [25], and Malay [28] validation studies showed lower floor effects, which might indicate poorer oral health in those populations [31]. Ceiling effects were not observed in other studies [22, 28, 29, 31] and were only minimally reported in the clinic sample in the current study.

Convergent validity of the A-ECOHIS was established. Caregivers who reported poor general oral health of their children had higher A-ECOHIS scores, which indicated worse OHRQoL. Unexpectedly, caregivers who reported good general oral health had slightly higher A-ECOHIS scores than did those who reported it to be fair. This could have been due to the subjectivity of the options, especially in two consecutive categories. Overall, there was a significant correlation between the global question and each of the total, child and family A-ECOHIS scores, as reported in other validation studies [22–24, 30, 31]. The Spearman correlation coefficient that we calculated for the total A-ECOHIS score with the global question was higher than that reported in the French study (−0.20) [23], but not the Turkish (0.68) [31] or Lithuanian (0.72) [29] studies.

As demonstrated in other ECOHIS validation studies [22, 24, 25, 27–29, 31], the A-ECOHIS was able to discriminate between children affected and not affected by oral disease. The total A-ECOHIS score and those of each section of the questionnaire were significantly higher among children with higher dmft scores compared with those with lower scores. The clinic sample, which we hypothesized would have a poorer oral health status than the community (school) sample, had significantly higher A-ECOHIS scores on all sections of the scale, as observed in other studies [23, 30]. This also indicated that caregivers are able to respond accurately regarding their child’s OHRQoL based on the child’s visible oral health condition [22, 42, 43].

The A-ECOHIS child and family section scores in this study were significantly correlated, suggesting that the scale is related to the concept it is intended to measure [22, 31], with a correlation coefficient that was within the same range as some reports (0.54 – 0.68) [23, 24, 29, 31], but higher than that of the ECOHIS development study (0.36) [22]. The inter-item correlations observed in this study also fell within the range of correlations observed in other studies (0.005 to 0.8) [23, 28–31].

Cronbach’s alpha values greater than 0.8, as were calculated for the A-ECOHIS, indicate excellent internal reliability [25, 44]. The values reported for the child section ranged from 0.79 to 0.92, in reports of the French and Turkish language versions, respectively [22–25, 27, 29–31]; in the family section, the values ranged from 0.65 to 0.95 in the Brazilian and original ECOHIS studies, respectively [22]. In this study, Cronbach’s alpha estimated for the A-ECOHIS as a whole was similar to that estimated in Brazilian and Lithuanian studies (0.86) [27, 29], and slightly higher than those of the French (0.82) [23] and Malay studies (0.83) [28], but lower than those of other studies (0.91 to 0.99) [24, 25, 31].

The test-retest reliability sample showed the stability in responses to the questionnaire. The ICC values for the child section and the total questionnaire fell within the ranges observed in other studies: 0.83 to 0.98 for the child section [23, 24, 27, 31] and 0.82 to 0.98 for the total questionnaire [22, 23, 27–31]. The family section ICC score in this study (0.67) was lower than that of most of the other studies (0.81 to 0.97) [23, 24, 27, 31]. The test-retest reliability of the Chinese study demonstrated the lowest ICC scores, at 0.64, 0.44 and 0.64 for the child, family sections and total questionnaire, respectively, which might be attributable to their small sample (*n* = 21) [25].

Missing values in validation studies can indicate a lack of comprehensibility or the irrelevance of items [23]. Missing responses in this study of the A-ECOHIS were infrequent, ranging from 0.2 to 5.5% in the community sample and not exceeding 2.8% in the clinic sample, which is less than what was reported previously [41]. This is a good indication of the comprehensibility and relevance of the A-ECOHIS items [23]. Adding “don’t know” options to questionnaires helps subjects answer questions thereby avoiding missing data [23]. Nevertheless, some of our respondents left some questions blank. The highest percentages of missing values were in the “financial impact” (5.5%) and “taking time off work” (4.7%) questions. It is possible that some of the caregivers were mothers unemployed outside of the home who did not have a full picture of the spouse’s work status or the family’s financial situation. Additionally, respondents may have felt embarrassed by or unwilling to expose their financial difficulties. Indeed, only a small percentage of subjects reported not having enough money, although it seems reasonable to assume that most of the patients who choose to visit the free clinics of King Abdulaziz University are financially insecure. The “don’t know” responses did not exceed 0.2% of the community-based sample and ranged from 0.8 to 2.1% of the clinic-based sample. This was much lower than the response rate for “don’t know” in the original study [22], and the French [23] and the Brazilian [24] validation studies.

A limitation of this study is that some factors hindered our planned data collection scheme; namely, the number and level of cooperation of public schools in Jeddah was very low. Furthermore, the fieldtrips coincided with the 2014 Middle-Eastern respiratory syndrome coronavirus outbreak crisis in Saudi Arabia. Many schools refused visits from healthcare providers because they were worried about the spread of infection from contaminated dentists. However, it should be noted that, in validation studies, it is acceptable to select samples based on validation needs [23, 45].

A family’s socioeconomic status (SES) can affect the caregiver’s perceptions regarding their child’s oral health [24, 26, 46] and is related to oral health conditions [47]. The discriminant validity results in this study, which compared the A-ECOHIS scores between the community and clinic samples, could have been influenced by the families’ SES differences. These differences were not accounted for because socioeconomic information was only collected for the clinic sample. Despite the potential socioeconomic differences between the samples, when an objective measure of oral health status (dmft score) was used to differentiate subjects based on their oral health status, the A-ECOHIS scale still exhibited discriminant validity.

Due to the very small number of public preschools in Jeddah, 81% of the school sample participants attended private preschools; however, Al Algili et al. demonstrated that school type is not always a good indicator of SES in this city [47]. Due to the shortage of public preschools in Jeddah, some caregivers who cannot easily afford it will enroll their children in lower cost private preschools.

Although there are many dialects of Arabic, the formal Arabic language, which is understood and read by all Arabic-speaking populations, was used in this version of the questionnaire. The questionnaire testing in our study was not restricted to the Saudi population; 50% our clinic-based sample and 37% of the community sample were from other Arabic nationalities. Therefore, it might have potential for use by other Arabic-speaking populations.

The strengths of this study are worth mentioning. The sample size was adequate for the analyses and was larger than most of the other ECOHIS validation studies [22, 23, 25–28, 30, 31]. Furthermore, the recruitment of two samples enabled the testing of more psychometric properties than studies comprised of one sample. Finally, the samples included all age groups covered by this scale.

## Conclusion

The A-ECOHIS performed well on all psychometric tests to which it was applied. It demonstrated convergent validity, discriminant validity, internal consistency, and test-retest reliability. Therefore, it is a valid and reliable instrument to use for Arabic-speaking caregivers of preschool-age children.

## Abbreviations

A-ECOHIS: Arabic Version of the Early Childhood Oral Health Impact Scale
CI: Confidence interval
dmft: Decayed missed and filled teeth
ECOHIS: Early Childhood Oral Health Impact Scale
ICC: Intra-class correlation coefficient
OHRQoL: Oral health-related quality of life
